# QTL Mapping of Trichome Traits and Analysis of Candidate Genes in Leaves of Wheat (*Triticum aestivum* L.)

**DOI:** 10.3390/genes15010042

**Published:** 2023-12-27

**Authors:** Hua Fan, Jianchao Xu, Dan Ao, Tianxiang Jia, Yugang Shi, Ning Li, Ruilian Jing, Daizhen Sun

**Affiliations:** 1 College of Agronomy, Shanxi Agricultural University, Taigu, Jinzhong 030800, China; jeanafan@163.com (H.F.); xjclike@163.com (J.X.); 18198321035@163.com (D.A.); jtt735982847@163.com (T.J.); shiyugang0804@126.com (Y.S.); 13159862006@163.com (N.L.); 2 Experimental Teaching Center, Shanxi Agricultural University, Taigu, Jinzhong 030800, China; 3 Institute of Crop Science, Chinese Academy of Agricultural Sciences, Beijing 100000, China; jingruilian@caas.cn

**Keywords:** mapping, QTL, wheat, trichome traits, candidate genes

## Abstract

Trichome plays an important role in heat dissipation, cold resistance, water absorption, protection of leaves from mechanical damage, and direct exposure to ultraviolet rays. It also plays an important role in the photosynthesis, transpiration, and respiration of plants. However, the genetic basis of trichome traits is not fully understood in wheat. In this study, wheat DH population (Hanxuan 10 × Lumai 14) was used to map quantitative trait loci (QTL) for trichome traits in different parts of flag leaf at 10 days after anther with growing in Zhao County, Hebei Province, and Taigu County, Shanxi Province, respectively. The results showed that trichome density (TD) was leaf center > leaf tip > leaf base and near vein > middle > edge, respectively, in both environments. The trichome length (TL) was leaf tip > leaf center > leaf base and edge > middle > near vein. Significant phenotypic positive correlations were observed between the trichome-related traits of different parts. A total of 83 QTLs for trichome-related traits were mapped onto 18 chromosomes, and each one accounted for 2.41 to 27.99% of the phenotypic variations. Two QTL hotspots were detected in two marker intervals: AX-95232910~AX-95658735 on 3A and AX-94850949~AX-109507404 on 7D. Six possible candidate genes (*TraesCS3A02G406000*, *TraesCS3A02G414900*, *TraesCS3A02G440900*, *TraesCS7D02G145200*, *TraesCS7D02G149200*, and *TraesCS7D02G152400*) for trichome-related traits of wheat leaves were screened out according to their predicted expression levels in wheat leaves. The expression of these genes may be induced by a variety of abiotic stresses. The results provide the basis for further validation and functional characterization of the candidate genes.

## 1. Introduction

Trichome is a unique hairy structure appendage developed from plant epidermal cells [1,2]. The density and size are determined by the stage of growth and development as well as its location [3]. Trichome not only provides a natural physical barrier for plants, reducing damage from harmful organisms, freezing, UV radiation, and mechanical injury, but it also helps to minimize water loss and excessive accumulation and dissipation of heat within the plant [3,4,5,6,7]. At the same time, trichomes can synthesize, store, and secrete many important substances, working in conjunction with the pores, keratin, and wax on the surface to fulfill various protective functions [8,9,10,11]. Trichomes play a crucial role in the photosynthesis, transpiration, and respiration processes of plants [12]. In order to adapt to the growing environment, trichomes respond to abiotic environmental factors such as salt, drought, altitude, and light [13,14,15].

At present, many reports on genetic analysis of trichomes have focused on Arabidopsis, cucumber, soybean, cotton, Artemisia annua, tomato, corn, rice, and so on. Some key genes for the development of the trichome in the model plant Arabidopsis have been cloned, and the regulatory mechanism of trichome development has been gradually uncovered. The major regulatory genes known are transcription factors such as *GL1* [16], *TTG1* [17,18], *GL3* [19,20], *EGL3* [21], *GL2* [22], *TRY* [23], *CPC* [24], and *ETC1* [25] et al. Vernoud et al. (2009) [26] have separated some *GLOSSY* genes from maize associated with trichome and wax synthesis. The known rice genes mainly include *OsWOX3B* (*GLR1*, *NUDA*/*GL-1/dep*, *GL5*) [27,28,29,30], *GL6* (*HL6*) [31,32,33], *glr2* [34], *GLR3* (*OsSPL10*) [35,36], *gl1* [37,38], *GLL* [39], etc. Mutations of these genes often lead to the reduction or loss of rice leaf and glume surface trichomes. The trichome-related genes cloned in cucumber include *TRIL* and *CsGL1*, both of which encode proteins that contain the Leucine zipper domain and play an important regulatory role in the initiation and formation of trichome [40,41,42]. The genes in cotton, *GoPGF* [41], and *GhMYB2A* [43] regulate the initiation of trichome and fiber formation. Du et al. (2009) [44] mapped two QTLs that control leaf trichome density in soybeans, *qtdD1b-2* and *qtdH-2*. The contribution of qtuH-2 to phenotypic variance was estimated to be 31.81% by molecular linkage groups and 29.4% by multiple interval mapping. Xing et al. (2013) [45] conducted a QTL analysis on the density and length of leaf pubescence in soybean by using two recombinant inbred lines, and they detected two major QTL, *PD1-1* and *PD12-1*, associated with the density of leaf pubescence; the QTL contributed >20% to the phenotypic variance.

Because of the characteristics of the wheat genome, research on trichomes has been slower than that on other crops. Ringlund et al. (1968) [46] considered trichome traits in the leaves of cultivated wheat to be quantitative genetic traits. Maystrenko et al. (1976) [47] reported that the leaf trichomes of wheat seedlings are controlled by the major gene Hl1 located on chromosome 4B, and this gene was mapped to the short arm of chromosome 4B by Arbuzova et al. (1996) [48] by using telomere analysis. Taketa et al. (2002) [49] performed haplotype and telomere analyses and found another major gene that controls leaf trichomes on chromosome 7BS. Previous studies have also reported the mapping of trichome genes in the non-leaf organs of wheat. Maystrenko et al. (1992) [50] found that Pa controls auricle trichomes, with only a 30 cM gap between *Hl1* and *Pa*. In addition, Blanco et al. (1998) [51] and Khlestkina (2002) [52] indicated that the trichomes on wheat glumes are regulated by the dominant gene *Hg* on the short arm of chromosome 1A. Furthermore, Love et al. (1924) [53] and Gaines (1926) [54] found that the dominant gene *Hn* that controls the trichomes on stem internodes is linked to the suppressor gene *B1* of awn, and Sourdille et al. (2002) [55] mapped *Hn* to the long arm of chromosome 5A. Wan et al. (2015) [56] detected a major QTL for leaf sheath hairiness on 4DL in wheat, and the locus was significantly and positively correlated with some yield characteristics. Luo et al. (2020) [57] described two pairs of NILs for hairy glumes in common wheat. Chen Zhitong et al. (2021) [58] detected a major QTL conditioning trichome length and density on chromosome arm 4BL and developed near-isogenic lines targeting this locus in bread wheat.

In this study, trichome density and length in different parts of leaves in rain-fed and irrigation environments were calculated. By using a high-density linkage genetic map, the QTLs of trichome-related traits and candidate genes will provide insights for studying the molecular mechanism of trichome development in the wheat leaf.

## 2. Materials and Methods

### 2.1. Materials

#### 2.1.1. Test Material

A wheat double haploid (DH) population, including 150 lines that were derived from a cross between Hanxuan 10 and Lumai 14, was used as test material in this study. All the DH lines and parents were grown in Zhao County (Heibei Province) and Taigu (Shanxi Province), China, in September 2019. For the experiment, a randomized block design was used with three repetitions. Wheat seeds were dibbled in 2 m rows, with 40 seeds per row and two rows per plot. In Zhao County, plants were irrigated before overwintering and at reviving, jointing, and mid-filling stages, with a total irrigation amount of 600 m^3^/hm^2^. In Taigu, the soil was irrigated to maintain available water before sowing, and rain-fed plants were not irrigated throughout the growth period. Precipitation over the whole growth period of wheat was 569.7 mm in Zhao County and 189 mm in Taigu. Taigu environment was named rain-fed, and Zhao County was irrigation.

#### 2.1.2. Sample Collection and Preparation

On the day of flowering, the main stems of five random plants with uniform growth were selected and marked. Ten days after flowering, the entire blade of flag leaves was cut and immediately placed in a pre-cooled 3% glutaraldehyde fixative solution (in 0.1 M phosphate-buffered saline [PBS], Ph = 7.0) and stored in a refrigerator at 4 °C. The fixed leaves were washed three times with 0.1 M PBS, and samples were cut from the leaf tip, leaf center, and leaf base. Then, the samples were dehydrated with a gradient ethanol series of 30%, 50%, 70%, 80%, 90%, and 100% (twice) for 15 min each, followed by 75% tert-butanol (in ethanol solution) and 100% tert-butanol (twice) for 20 min each. The dehydrated samples were freeze-dried (JFD-320; JEOL Ltd., Akishima, Tokyo, Japan), stuck on stubs, and coated with platinum by using an ion sputtering device (JEM-6490LV; JEOL). The prepared samples were observed under a scanning electron microscope (JEM-6490LV; JEOL).

### 2.2. Methods

#### 2.2.1. Trichome Trait Measurement and Data Analysis

Under the scanning electron microscope, three fields of view were selected from different parts (edge, middle [between leaf edge and main vein], and near vein) at the tip, center, and base of each leaf. A total of 27 fields of view were observed, and images were obtained at 100× magnitude. The Smile View software (Scanning electron microscopy analysis software of Japan Electron Optics Laboratory, JEOL) was used to measure the number of trichomes (then converted to trichome density, trichomes/mm^2^) and trichome length (μm). Correlation analysis between trichome traits of the wheat leaves was performed using SPSS v17.0 (SPSS Inc., Chicago, IL, USA).

#### 2.2.2. QTL Mapping

The genetic map of the DH population was constructed by Jing Ruilian’s team at the Institute of Crop Science, Chinese Academy of Agricultural Sciences.

The map was constructed using a 660 K single-nucleotide polymorphism (SNP) array and simple sequence repeat (SSR) markers. It contained a total of 30 linkage groups with 1854 markers, including 1630 SNP markers and 224 pairs of SSR markers. It spanned 4082.44 cM of 21 wheat chromosomes, with a mean distance of 2.20 cM between markers [59].

On the basis of the complete interval mapping method, QTL detection was performed using IciMapping v4.0 (http://www.isbreeding.net/ (accessed on 15 November 2022)). The limit of detection (LOD) threshold was set to 2.5 [60], and QTLs with overlapping confidence intervals were integrated. QTL naming adopted Q + trait abbreviation + chromosome. If multiple QTLs were detected on the same chromosome for a specific trait, the number 1, 2, or 3 was added after the chromosome in the QTL name.

#### 2.2.3. Prediction of Candidate Genes

Candidate genes in associated loci were predicted according to the reference genome sequence of ‘Chinese Spring’ wheat (IWGSC RefSeqv1.1), published by the International Wheat Genome Sequencing Consortium. Gene annotation was carried out by referring to the Ensembl Plants database (https://plants.ensembl.org/index.html (accessed on 9 October 2023)). We used a publicly available database, WheatOmics (http://wheatomics.sdau.edu.cn/ (accessed on 15 December 2023)) [61] (Ma et al., 2021), to obtain the expression profiles of all candidate genes.

## 3. Results

### 3.1. Distribution of Trichomes on Wheat Leaves

**Figure 1:**
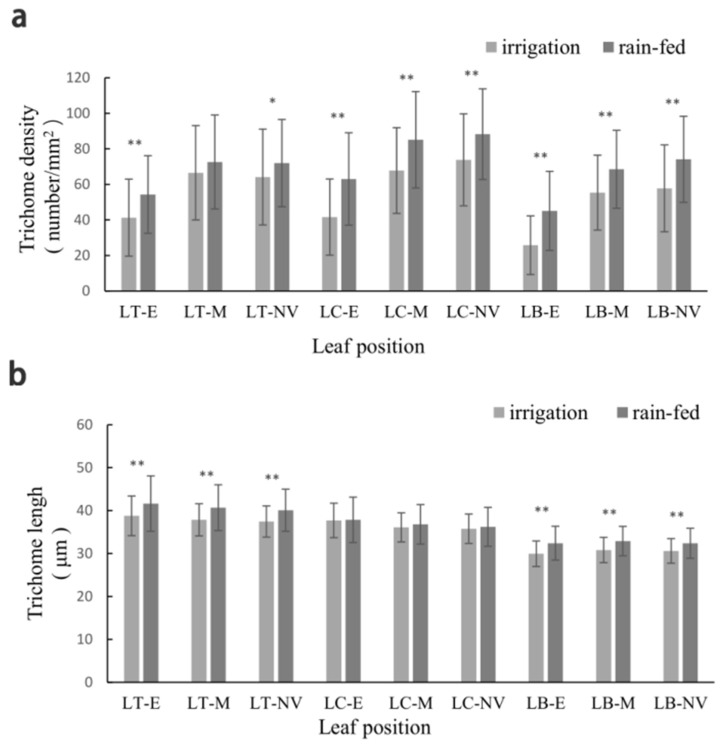
The change trend lines of TD (**a**) and TL (**b**) for DH lines at different parts under two environments. LT-E: Leaf Top–Edge; LT-M: Leaf Top-Middle; LT-NV: Leaf Top-Near Vein; LC-E: Leaf Central-Edge; LC-M: Leaf Central-Middle; LC-NV: Leaf Central-Near Vein; LB-E: Leaf Base-Edge; LB-M: Leaf Base-Middle; LB-NV: Leaf Base-Near Vein. * and ** represent significant levels at *p* = 0.05 and *p* = 0.01 in the same row. TD: Trichome Density.

The different parts of the wheat leaves were observed (Supplementary Figure S1). TD was in the following order across the two environments: leaf center > leaf tip > leaf base under the two environments (Table 1). Under irrigation conditions, a significant difference (*p* < 0.01) in TD was observed between the leaf center, or tip, and the leaf base. Under rain-fed conditions, a significant difference (*p* < 0.01) in TD was observed between the leaf center and leaf base or tip. TD was in the following order across the two environments: near vein > middle > edge. A significant difference (*p* < 0.01) in TD was observed between the near vein, middle, and edge of each leaf part (Table 1, Figure 1). And it was found that, except for the leaf tip, TD at different leaf parts was significantly higher (*p* < 0.01) under rain-fed conditions than under irrigation. In particular, TD at the leaf base and edge was 20.47% and 40.37% higher under rain-fed conditions than under irrigation, respectively.

In both environments, TL varied in different parts of the wheat leaves (leaf tip > leaf center > leaf base), and the differences between various leaf parts were significant (*p* < 0.01). In each part of the leaves, TL varied at different parts (edge > middle > near vein), and the difference was significant between the edge and near vein (Table 1, Figure 1). TL tended to increase distinctly at different parts of wheat leaves under rain-fed conditions than under irrigation, and the differences were significant between the two environments (*p* < 0.01), except for the leaf center (Table 1).

### 3.2. Phenotypic Characteristics of Trichome-Related Traits in the DH Lines and Parents

The phenotypic values of TD and TL differed among the DH wheat lines and parents in the two environments. Under irrigation conditions, the male parent Lumai 14 had a higher TD than the female parent Hanxuan 10 at different parts of the wheat leaves, except for the leaf base. Under rain-fed conditions, Lumai 14 had a higher TD than Hanxuan 10 at all leaf parts. Furthermore, TL at different leaf parts was consistently higher for Hanxuan 10 than for Lumai 14 in both environments (Supplementary Table S1).

TD and TL of the DH lines were both higher under rain-fed conditions than under irrigation conditions. The mean values were between those of the two parents, but the range of variation showed remarkable bidirectional transgressive segregation. The coefficients of variation for TD ranged from 28.89% to 64.05%, and the coefficients of variation for TL ranged from 9.36% to 15.50%. Both TD and TL exhibited continuous variation, with less than 1 skewness and kurtosis (Supplementary Table S1), basically showing a normal distribution. The results indicate that TD and TL are quantitative traits controlled by polygenes.

### 3.3. Correlations of Trichome Traits in Wheat Leaves

**Figure 2:**
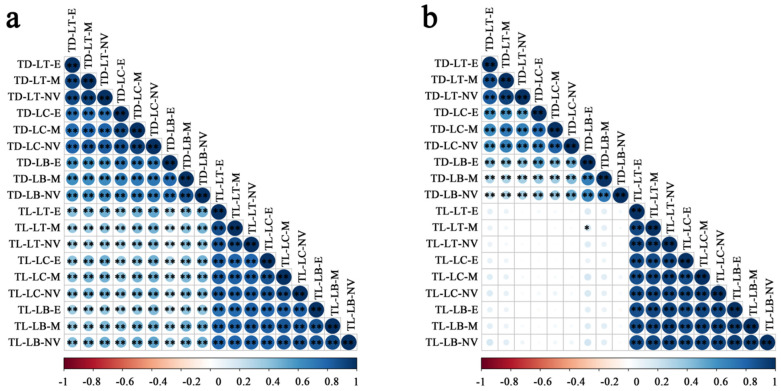
Correlation of trichome density and trichome length of wheat leaves under rain-fed (**a**) and irrigation (**b**) conditions. * and ** represent significant and extremely significant levels, respectively.

In both environments, TD and TL showed a significantly positive correlation for all parts of the leaves. Under the rain-fed condition, TD showed a significantly positive correlation with TL for all parts. Under the irrigation condition, only TD at the leaf base-edge and TL at the leaf top-middle were significantly phenotypically positively correlated (Figure 2b).

### 3.4. QTL for Trichome Density in Wheat Leaves

A total of 49 QTLs for TD were detected in two environments. The phenotypic variance ranged from 2.41 to 27.99%, and the LOD value ranged from 2.57 to 22.32. The QTLs were distributed on 15 chromosomes, including 1D, 2A, 2B, 2D, 3A, 3B, 3D, 4D, 5A, 5B, 6A, 6B, 6D, 7B, and 7D, respectively. A total of 23 QTLs were obtained under irrigation, and the other 26 were rainfed. A total of 18 QTLs with positive effects were derived from the female parent Hanxuan 10 and distributed on nine chromosomes (2A, 2B, 3D, 5B, 6A, 6B, 6D, 7B, and 7D). The remaining 31 QTLs with negative effects were obtained from the male parent, Lumai 14, and distributed on eight chromosomes (1D, 2A, 2D, 3A, 3B, 4D, 5A, and 6A).

A total of 8 QTLs were detected in both environments. Among these QTLs, *Qtd-3A-1* was detected at the leaf tip–middle, leaf base–edge, and leaf base–middle under rain-fed conditions, as well as the leaf tip–near vein under irrigation conditions, and was in the interval AX-95653062-AX-95235020 on chromosome 3A. *Qtd-3A-4* was detected at the leaf center–middle and leaf center–near vein under rain-fed and leaf tip–middle under irrigation conditions and was in the interval AX-95235020-AX-95658735l. *Qtd-3A-1* and *Qtd-3A-4* were located at adjacent marker intervals. *Qtd-3A-2* was identified at the leaf tip–near vein under rain-fed conditions as well as the leaf tip–edge and leaf center–near vein under irrigation conditions and was in the interval AX-95207768-AX-95679334. *Qtd-3A-3* was detected at the leaf center–edge and leaf base–near vein under rain-fed and leaf base–middle irrigation conditions and was in the interval AX-95232910-AX-95207768. *Qtd-3A-2* and *Qtd-3A-3* were located at adjacent marker intervals.

### 3.5. QTL for Trichome Length in Wheat Leaves

A total of 34 QTLs for TL were detected in two environments. The phenotypic variance ranged from 5.52 to 18.46%, and the LOD value ranged from 2.50 to 7.53. The QTLs were distributed on 12 chromosomes, including 1A, 1B, 2A, 2B, 2D, 3B, 3D, 4A, 6B, 6D, 7B, and 7D, respectively. Approximately 16 QTLs were obtained under irrigation, and the other 18 were rainfed. A total of 29 QTLs with positive effects were derived from the female parent Hanxuan 10 and distributed on nine chromosomes (2A, 2B, 3B, 3D, 4A, 6B, 6D, 7B, and 7D). The remaining 5 QTLs with negative effects were obtained from the male parent, Lumai 14, and distributed on four chromosomes (1A, 1B, 2D, and 7B).

**Figure 3:**
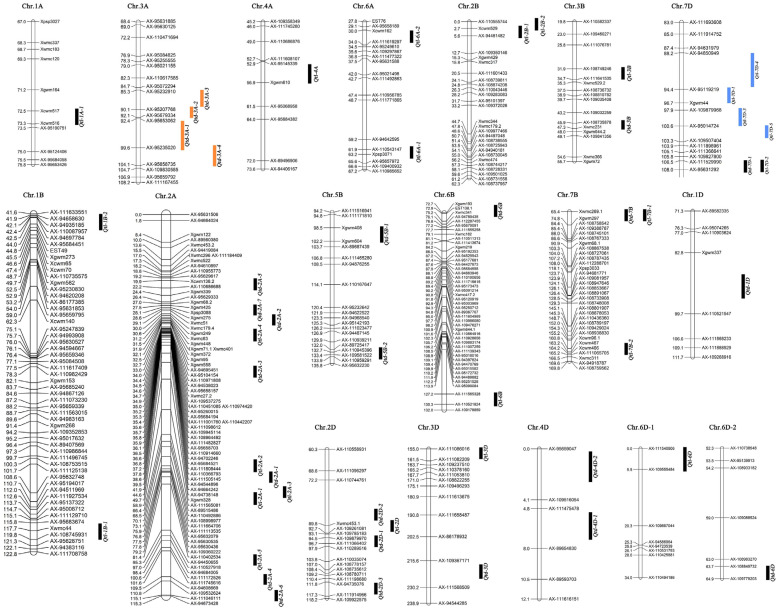
Distributions of identified QTL for trichome traits on genetic linkage maps, The yellow lines are the QTL of trichome density, and the blue lines are the QTL of trichome length.

A total of 12 QTLs were detected in both environments. Among these QTLs, *Qtl-7D-1* was detected at not only the leaf tip–middle and leaf base–middle under irrigation but also the leaf tip–middle and leaf center–near vein under rainfed conditions and was in the interva AX-95119219–Xgwm44 on chromosome 7D. *Qtl-7D-4* was detected at the leaf center–near vein under irrigation and leaf tip–edge under rainfed conditions, and was in the interva AX-94850949–AX-95119219. *Qtl-7D-1* and *Qtl-7D-4* were located at adjacent marker intervals. *Qtl-7D-3* was detected at the leaf center–middle under irrigation and leaf tip–near vein and leaf base–near vein under rainfed conditions and was in the interva AX-109879968–AX-95014724. *Qtl-7D-5* was detected at the leaf base–edge under irrigation and leaf center–edge and leaf base–middle under rainfed conditions, and was in the interva AX-95014724–AX-109507404. *Qtl-7D-3* and *Qtl-7D-5* were located at adjacent marker intervals (Figure 3).

### 3.6. The Prediction of Candidate Genes

**Figure 4:**
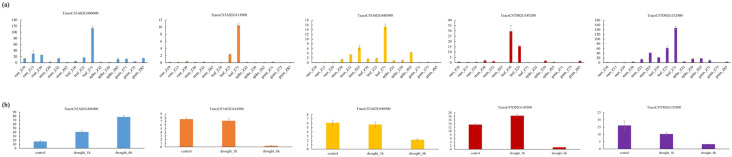
Expression of candidate genes in various tissues (**a**). Expression of candidate genes under drought stress (**b**).

According to the reference genome sequence of ‘Chinese Spring Wheat’ (IWGSC RefSeqv1.1) published by the International Wheat Genome Sequencing Consortium, 285 genes were found in the interval of AX-95232910~AX-95658735 (18.8 cM) on chromosome 3A, and 150 genes were found in the interval of AX-94850949~AX-109507404 (15.1 cM) on chromosome 7D, and gene annotation was performed with reference to the Ensembl Plants database. WheatOmics was used to predict the expression of these genes in different tissues of Chinese Spring. We found that five genes (*TraesCS3A02G406000, TraesCS3A02G414900*, *TraesCS3A02G440900*, and *TraesCS7D02G149200, TraesCS7D02G152400*) were highly expressed in leaves, suggesting that these trichomes may have the function of regulating leaf trichome traits (Figure 4).

Then, the expression levels of these five genes under drought stress were predicted. The results showed that drought stress induced the expression of these genes. For example, the expression of *TraesCS3A02G406000* was significantly increased after drought stress, and the expression of *TraesCS3A02G414900, TraesCS3A02G440900, TraesCS7D02G149200,* and *TraesCS7D02G152400* was significantly decreased by drought induction (Figure 4).

## 4. Discussion

### 4.1. Genetic Effects of Trichome-Related Traits

In this study, we found that TD and TL of wheat leaves varied with different parts on the flag leaves of wheat, and the trend of each trait was similar across two different environments. These results indicated that trichome TD and TL were relatively stable traits in wheat that were less affected by the environment.

Tanksley (1996) [62] indicated that a major QTL refers to a QTL that explains >10% of the total phenotypic variance. In the present study, we detected 49 QTLs with additive effects on TD in the flag leaves of wheat, 12 of which each explained >10% of the phenotypic variance. In addition, 34 QTLs with additive effects on TL were detected in wheat, 11 of which each explained >10% of the phenotypic variance. According to these results, TD and TL in wheat were quantitative traits controlled by a combination of major and minor genes.

### 4.2. Linkage and Pleiotroty of QTL Related to Trichome Traits in Wheat

Various studies have found that QTL for closely related traits may be located in the same or nearby parts of chromosomes [63,64]. In this study, we found that under both environments, significant phenotypic positive correlations were observed between the trichome densities of different parts and between the trichome lengths of different parts. And 4 TD QTLs, *Qtd-3A-1*, *Qtd-3A-2, Qtd-3A-3*, and *Qtd-3A-4*, for more than one part of wheat leaves were detected between AX-95232910 and AX-95658735 under the two environments, respectively (Table 2 and Figure 3). Approximately 4 TL QTLs (*Qtl-7D-1, Qtl-7D-3, Qtl-7D-4*, and *Qtl-7D-5*) were also conducted between AX-94850949 and AX-109507404, respectively (Table 3 and Figure 3). Moncada et al. (2001) [65] believed that such specific regions related to multiple traits may have linkage or epistatic effects. Therefore, TD or TL in the different parts of wheat leaves may be controlled by the same gene or linkage genes.

Pleiotropism has been found in the QTL mapping and GWAS of multiple crops [66,67]. In this study, TD QTL, *Qtd-7D-1,* and TL QTL, *Qtl-7D-2*, were detected between AX-111529990-AX-95631292 at leaf top- middle and leaf top-near vein under rain-fed conditions, respectively. *Qtd-7B* and *Qtl-7B-1* were detected in the interval Xwmc269.1-Xgwm297 at leaf top-edge and leaf central-edge under rain-fed conditions, respectively (Table 2 and Table 3). Therefore, pleiotropic sites controlling TD and TL of the trichome may be in the two regions.

### 4.3. Prediction of Candidate Genes Associated with Trichome Traits

In this study, 285 genes were found in the AX-95232910~AX-95658735 (18.8 cM) interval of chromosome 3A, and 150 genes were found in the AX-94850949~AX-109507404 (15.1 cM) interval of chromosome 7D. Five candidate genes (*TraesCS3A02G406000, TraesCS3A02G414900, TraesCS3A02G440900, TraesCS7D02G149200, TraesCS7D02G152400*) were screened according to their expression levels in different tissues of wheat Chinese spring varieties and after drought stress. These genes were mainly highly expressed in leaves and significantly induced by drought stress.

The candidate gene *TraesCS3A02G406000* encodes a NAC domain protein. It has been reported in wheat that knocking out the NAC gene (TaNAC071-A) reduces wheat drought tolerance [68]. Under drought conditions, the expression of *TraesCS3A02G406000* was significantly increased. *TraesCS3A02G414900* is specifically expressed in wheat leaves, and its function is annotated as a cysteine-rich receptor kinase. Cysteine-rich receptor-like kinases (CRKs) belong to a large family of receptor-like kinases (RLKs) containing duf26, which play a key role in immunity, abiotic stress response, and growth and development [69]. Flor Cristina Arellano-Villagómez et al. found that Arabidopsis cysteine-rich receptor-like protein kinase CRK33 affects stomatal density and drought tolerance [70]. The expression of the *TraesCS3A02G414900* gene was significantly decreased after drought stress. *TraesCS3A02G440900* encodes a sec14p-like phosphatidylinositol transfer family protein. The main PI (phosphatidylinositol)/PC (phosphatidylcholine) transfer protein Sec14p in yeast coordinates lipid metabolism and protein transport of the Golgi complex [71]. The expression level of the gene *TraesCS3A02G440900* was significantly reduced under drought conditions. *TraesCS7D02G149200* is highly expressed in wheat leaves, and its functional annotation is the Dirigent protein. Dirigent protein has stereoselectivity to phenoxy coupling reactions in plants, so it plays an important role in the biosynthesis of bioactive natural products [72]. After drought stress, the expression of the *TraesCS7D02G149200* gene was significantly reduced. The functional annotation of *TraesCS7D02G152400* is glutathione peroxidase. Glutathione peroxidase 1 (GPx1) is an important cellular antioxidant enzyme. GPx1 regulates the balance between essential and harmful reactive oxygen species levels [73]. The expression of the *TraesCS7D02G152400* gene was significantly reduced after drought stress.

The above five genes were not only specifically highly expressed in wheat leaves but also induced by drought stress. Studies have shown that wheat trichome traits have the effect of buffering direct sunlight and preventing water evaporation, thereby improving wheat drought resistance. In this study, these five candidate genes were significantly induced by drought stress. We speculated that these genes may also affect wheat drought tolerance by regulating wheat trichome traits. Therefore, in future studies, we will analyze whether these five genes have the function of regulating trichome traits through transgenic experiments, which will provide new insights into wheat drought tolerance research.

## 5. Conclusions

This study with 150 DH lines revealed that TD and TL were quantitatively inherited traits. Significant phenotypic positive correlations were observed between the trichome-related traits of different parts. A total of 83 QTLs were identified for TD and TL of wheat leaves in the two environments, which were distributed across 18 chromosomes and explained 2.41 to 27.99% of phenotypic variation. Two QTL hotspots were found in 3A and 7D. Five candidate genes were screened out, according to their expression levels in wheat leaves. The results provide the basis for further validation and functional characterization of the candidate genes.

## Figures and Tables

**Table 1:** Comparisons of trichome traits of DH lines and parents at different parts of the leaf under two environments.

Part	TD		TL	
	Irrigation	Rain-Fed	Irrigation	Rain-Fed
LT	57.076 Aa	61.745 Bb	37.967 Aa	40.754 ** Aa
LC	60.813 Aa	72.973 ** Aa	36.49 2 Bb	36.843 Bb
LB	46.218 Bb	55.680 ** Bb	30.276 Cc	32.634 ** Cc
E	35.785 Bb	50.231 ** Bb	35.380 Aa	37.264 ** Aa
M	62.535 Aa	70.075 ** Aa	34.816 Aab	36.717 ** Aab
NV	64.930 Aa	72.653 ** Aa	34.540 Ab	36.257 ** Ab

Note: The capital letter and small letter represent the difference at the 1% and 5% levels, respectively, in the same column; ** represent significant levels at *p* = 0.01 in the same row. TD: Trichome Density; TL: Trichome Length; LT: Leaf Top; LC: Leaf Central; LB: Leaf Base; E: Edge; M: Middle; NV: Near Vein.

**Table 2:** Additive-effect QTLs for trichome density under two environments.

QTL	Environment	Part	Positon ^(1)^	Flanking Marker	LOD ^(2)^	PVE (100%) ^(3)^	Add ^(4)^
*Qtd-2A-1*	Rain-fed	LT-E	38	AX-110366793-AX-111505145	9.75	17.94	−10.09
		LT-NV			5.45	14.97	−9.63
*Qtd-2A-2*	Irrigation	LT-E	29	Xgwm275-Xwmc51	7.58	15.66	9.36
*Qtd-2A-3*	Irrigation	LC-E	34	AX-94695451-AX-95104154	8.84	14.929	−11.36
		LC-M			15.65	21.83	−16.71
		LC-NV			22.32	27.993	−17.94
*Qtd-2A-4*	Irrigation	LC-E	101	AX-111172526-AX-111745616	4.84	7.689	8.15
*Qtd-2A-5*	Irrigation	LC-M	20	Xcwm138.2-AX-110686688	5.64	6.92	9.52
*Qtd-2A-6*	Irrigation	LC-M	115	AX-109532624-AX-111046111	3.85	4.43	7.54
*Qtd-2A-7*	Irrigation	LC-NV	28	Xgwm425-Xpsp3088	8.79	9.923	10.68
*Qtd-3A-1*	Rain-fed	LT-M	96	AX-95653062-AX-95235020	4.69	12.37	−8.94
		LB-E	99		2.98	9.06	−5.05
		LB-M			3.30	9.24	−6.43
	Irrigation	LT-NV	96		5.10	11.93	−8.25
*Qtd-3A-2*	Rain-fed	LT-NV	91	AX-95207768-AX-95679334	6.18	16.67	−10.22
	Irrigation	LT-E	92		2.90	5.34	−5.52
		LC-NV			5.12	4.69	−7.40
*Qtd-3A-3*	Rain-fed	LC-E	90	AX-95232910-AX-95207768	4.31	9.20	−6.55
		LB-NV			4.427	10.12	−8.19
	Irrigation	LB-M	89		4.73	11.17	−8.16
*Qtd-3A-4*	Rain-fed	LC-M	100	AX-95235020-AX-95658735	4.09	10.15	−6.67
		LC-NV			3.76	10.39	−7.78
	Irrigation	LT-M			4.97	10.37	−8.26
*Qtd-3A-5*	Irrigation	LB-E	83	AX-110617585-AX-95072294	4.92	11.68	−8.07
*Qtd-5A-1*	Irrigation	LT-M	0	AX-109541070-AX-95630232	3.65	7.29	−6.95
*Qtd-5A-2*	Irrigation	LT-NV	20	AX-95659236-AX-109921026	3.07	6.89	−6.37
*Qtd-6A-1*	Rain-fed	LB-M	63	AX-110543147-Xpsp3071	2.57	7.34	5.90
*Qtd-6A-2*	Irrigation	LC-E	33	Xcwm162-AX-111619297	5.39	8.98	−9.28
*Qtd-2B-1*	Rain-fed	LC-NV	3	Xcwm529-AX-94481482	4.09	11.28	8.37
*Qtd-2B-2*	Irrigation	LT-M	2	AX-110555744-Xcwm529	2.66	5.86	6.39
*Qtd-3B*	Rain-fed	LT-E	47	AX-108735878-Xwmc231	4.93	9.56	−7.359
*Qtd-5B-1*	Rain-fed	LB-NV	99	Xgwm408-Xgwm604	2.577	5.97	6.27
*Qtd-5B-2*	Irrigation	LC-NV	133	AX-110945396-AX-109581522	2.65	2.41	5.30
*Qtd-5B-3*	Irrigation	LB-NV	249	AX-109455033-AX-108853192	2.76	8.60	7.08
*Qtd-6B*	Rain-fed	LT-E	75	EST138.1-Xwmc341	3.18	5.19	6.559
*Qtd-7B*	Rain-fed	LT-E	66	Xwmc269.1-Xgwm297	4.63	7.92	6.70
*Qtd-1D*	Irrigation	LC-NV	86	Xgwm337-AX-110521547	3.19	3.223	−6.08
*Qtd-2D-1*	Rain-fed	LC-E	95	AX-109879970-AX-111066402	6.93	15.24	−8.42
*Qtd-2D-2*	Rain-fed	LB-NV	89	AX-110744761-Xwmc453.1	4.47	10.64	−8.45
*Qtd-2D-3*	Irrigation	LB-M	116	AX-94735076-AX-111914966	3.53	8.13	−6.94
*Qtd-3D*	Rain-fed	LC-M	216	AX-109367171-AX-111568509	3.09	7.55	5.76
		LC-NV			3.51	9.60	7.49
*Qtd-4D-1*	Irrigation	LC-E	5	AX-111475478-AX-89654830	4.94	7.86	−8.25
		LC-M	7		5.77	6.913	−9.39
		LC-NV	8		6.67	6.42	−8.58
*Qtd-4D-2*	Irrigation	LB-E	2	AX-95659047-AX-109516054	4.27	9.99	−7.47
*Qtd-6D*	Rain-fed	LT-E	64	AX-108849732-AX-109779203	2.60	4.07	4.80
*Qtd-7D-1*	Rain-fed	LT-M	108	AX-111529990-AX-95631292	2.57	6.24	6.35
*Qtd-7D-2*	Rain-fed	LC-M	101	AX-95014724-AX-109507404	5.31	13.49	7.77

Note: (1) Position (cM) represents the distance to the first marker in the linkage group; (2) LOD: LOD value of each QTL; (3) PVE indicates the phenotypic variance explained by additive QTL; (4) Add represents the additive effect, positive value indicates the Hanxuan 10 allele having a positive effect on the trait, and a negative value represents the Lumai 14 allele having a positive effect.

**Table 3:** Additive effect QTLs for trichome length under two environments.

QTL	Environment	Part	Position ^(1)^	Flanking Marker	LOD ^(2)^	PVE (100%) ^(3)^	Add ^(4)^
*Qtl-1A*	Rain-fed	LC-E	73	Xcwm517-Xcwm516	2.50	6.03	−1.07
*Qtl-2A-1*	Rain-fed	LT-E	45	AX-94738148-Xgwm328	4.48	12.54	1.588
		LT-M	46		4.66	12.23	1.29
*Qtl-2A-2*	Rain-fed	LT-NV	37	AX-95684521-AX-111808444	3.18	8.53	1.05
*Qtl-2A-3*	Irrigation	LT-E	43	AX-94664242-AX-94738148	7.53	18.46	2.83
		LT-M	44		3.56	9.53	1.65
*Qtl-2A-4*	Irrigation	LT-NV	31	Xgwm249-Xwmc63	2.78	7.21	1.29
*Qtl-2A-5*	Irrigation	LB-E	95	AX-110402534-AX-94450655	2.57	6.66	1.04
*Qtl-4A*	Rain-fed	LC-M	53	AX-95145339-Xgwm610	3.68	8.09	1.00
*Qtl-1B-1*	Rain-fed	LT-E	118	Xwmc44-AX-108745931	2.95	8.16	−1.28
*Qtl-1B-2*	Rain-fed	LC-NV	42	AX-94658630-AX-94935185	2.54	9.18	−0.89
*Qtl-2B-1*	Rain-fed	LC-E	54	AX-108725943-AX-94940181	2.90	6.51	1.12
*Qtl-2B-2*	Rain-fed	LC-M	58	AX-108744217-AX-108728331	2.57	5.52	0.84
*Qtl-3B*	Irrigation	LT-M	34	AX-108749246-AX-111641535	2.91	7.23	1.44
*Qtl-6B*	Irrigation	LT-E	128	AX-111565328-AX-110521824	3.13	6.63	1.70
*Qtl-7B-1*	Rain-fed	LC-E	74	Xwmc269.1-Xgwm297	4.02	10.37	−1.39
*Qtl-7B-2*	Irrigation	LB-E	165	Xcwm466-AX-111065705	4.13	11.30	1.35
*Qtl-7B-3*	Irrigation	LB-NV	145	AX-108748008-AX-108801907	3.50	11.24	1.03
*Qtl-2D*	Irrigation	LC-M	90	Xwmc453.1-AX-109261081	3.39	10.79	−1.48
*Qtl-3D*	Irrigation	LB-M	161	AX-111086016-AX-111082209	3.96	8.49	1.08
*Qtl-6D*	Irrigation	LB-M	0	AX-111540806-AX-109555484	3.46	7.24	1.01
*Qtl-7D-1*	Rain-fed	LT-M	96	AX-95119219-Xgwm44	6.09	16.53	1.51
		LB-M			3.09	9.48	1.20
	Irrigation	LT-M	96		4.27	10.94	1.78
		LC-NV	95		5.66	16.01	1.83
*Qtl-7D-2*	Rain-fed	LT-NV	108	AX-111529990-AX-95631292	3.91	10.18	1.15
*Qtl-7D-3*	Rain-fed	LC-M	98	AX-109879968-AX-95014724	7.48	17.43	1.48
	Irrigation	LT-NV	100		6.43	17.70	2.05
		LB-NV	98		4.53	14.77	1.19
*Qtl-7D-4*	Rain-fed	LC-NV	94	AX-94850949-AX-95119219	3.88	14.14	1.12
	Irrigation	LT-E	93		4.81	10.60	2.17
*Qtl-7D-5*	Rain-fed	LB-E	103	AX-95014724-AX-109507404	2.67	8.34	1.09
	Irrigation	LC-E	101		2.99	9.13	1.60
		LB-M	103		4.94	10.65	1.21

Note: (1) Position (cM) represents the distance to the first marker in the linkage group; (2) LOD: LOD value of each QTL; (3) PVE indicates the phenotypic variance explained by additive QTL; (4) Add represents the additive effect, positive value indicates the Hanxuan 10 allele having a positive effect on the trait, and a negative value represents the Lumai 14 allele having a positive effect. LT-E: Leaf Top-Edge; LT-M: Leaf Top-Middle; LT-NV: Leaf Top-Near Vein; LC-E: Leaf Central-Edge; LC-M: Leaf Central-Middle; LC-NV: Leaf Central-Near Vein; LB-E: Leaf Base-Edge; LB-M: Leaf Base-Middle; LB-NV: Leaf Base-Near Vein.

## Data Availability

Data are contained within the article and Supplementary Materials.

## Supplementary Materials

The following supporting information can be downloaded at: https://www.mdpi.com/article/10.3390/genes15010042/s1; Figure S1: Electron microscope images of trichome traits under full water (WW) and drought conditions (DS); Table S1: Phenotypic variation of trichome traits of DH lines and their parents at different part under two environments.
