# Supplementary material for: QTL Mapping of Trichome Traits and Analysis of Candidate Genes in Leaves of Wheat (Triticum aestivum L.)
Source: Genes (Basel). 2023 Dec 27;15(1):42. doi: 10.3390/genes15010042 (PMC10815787; doi:10.3390/genes15010042)
Supplement: Supplementary file 1 [file genes-15-00042-s001.zip › Supplementary Table 1.pdf]

Supplementary Table 1 Phenotypic variation of trichome traits of DH lines and their parents at different part under two environments

Trait	Part	Environment	Parent		DH line						
			Hanhuan10	Lumai14	Mean	SD	Skewness	Kurtosis	Minimum	Maximum	CV（%）
TD	LT-E	Irrigation	30.26	43.13	41.28	21.67	0.816	0.052	4.16	109.94	52.50
		Rain-fed	46.36	65.52	54.32	21.85	0.594	0.561	15.27	139.09	40.22
	LT-M	Irrigation	49.60	61.92	66.54	26.55	0.298	-0.576	15.82	132.43	39.90
		Rain-fed	56.91	90.78	72.63	26.41	0.589	0.555	19.43	159.91	36.36
	LT-NV	Irrigation	45.44	59.10	64.16	26.99	0.529	-0.352	16.66	135.76	42.07
		Rain-fed	60.62	78.71	72.01	24.55	0.395	-0.018	18.32	148.81	34.09
	LC-E	Irrigation	24.25	38.53	41.58	21.45	0.838	0.602	5.83	116.33	51.59
		Rain-fed	34.15	67.05	63.04	26.02	0.316	-0.175	8.33	136.59	41.27
	LC-M	Irrigation	49.60	65.34	67.79	24.11	0.284	-0.639	21.93	126.32	35.57
		Rain-fed	56.08	85.51	85.11	27.16	0.590	0.251	33.32	170.74	31.91
	LC-NV	Irrigation	62.37	64.91	73.85	25.88	0.244	-0.204	16.25	145.48	35.05
		Rain-fed	60.52	94.39	88.27	25.51	0.589	0.090	44.98	169.08	28.89
	LB-E	Irrigation	22.03	5.27	25.78	16.51	0.695	-0.194	2.50	73.85	64.05
		Rain-fed	39.98	40.40	45.13	22.25	0.474	0.381	5.00	175.74	49.31
	LB-M	Irrigation	53.21	22.49	55.39	21.08	0.594	0.746	10.55	130.21	38.06
		Rain-fed	71.63	72.46	68.53	21.91	0.481	0.008	27.49	134.93	31.97
	LB-NV	Irrigation	56.45	27.67	57.79	24.46	0.649	0.397	9.99	134.65	42.32
		Rain-fed	83.29	82.46	74.13	24.22	0.528	0.598	19.99	157.42	32.68

Supplementary Table 1 (continued)

Trait	Part	Environment	Parent		DH line						
			Hanhuan10	Lumai14	Mean	SD	Skewness	Kurtosis	Minimum	Maximum	CV (%)
TL	LT-E	Irrigation	46.13	33.37	38.78	4.61	0.626	0.844	27.00	53.40	11.90
		Rain-fed	46.93	37.70	41.64	6.45	0.960	1.298	31.40	67.80	15.50
	LT-M	Irrigation	48.37	34.90	37.86	3.75	0.338	0.433	27.50	48.10	9.90
		Rain-fed	45.80	40.80	40.69	5.34	0.662	0.705	30.00	59.60	13.12
	LT-NV	Irrigation	44.53	35.07	37.47	3.63	0.364	0.906	27.90	48.60	9.68
		Rain-fed	45.07	39.30	40.08	4.90	0.274	0.359	27.00	55.80	12.24
	LC-E	Irrigation	40.53	33.80	37.71	4.02	0.226	0.531	25.10	49.10	10.66
		Rain-fed	44.77	34.17	37.85	5.28	0.186	-0.442	26.80	52.20	13.95
	LC-M	Irrigation	39.67	36.13	36.09	3.39	0.745	0.664	29.80	47.10	9.41
		Rain-fed	42.80	31.90	36.80	4.60	0.263	-0.115	25.60	48.80	12.49
	LC-NV	Irrigation	39.57	36.07	35.78	3.42	0.528	0.883	27.00	48.10	9.57
		Rain-fed	40.80	31.67	36.23	4.52	0.292	-0.145	25.80	47.80	12.48
	LB-E	Irrigation	28.53	28.88	29.95	2.97	0.124	0.567	21.00	39.20	9.91
		Rain-fed	32.73	31.27	32.42	3.94	0.144	-0.360	22.40	42.80	12.14
	LB-M	Irrigation	29.23	28.32	30.83	2.94	0.794	0.960	24.90	40.90	9.55
		Rain-fed	34.20	31.27	32.90	3.43	0.270	-0.139	24.60	41.00	10.41
	LB-NV	Irrigation	31.20	28.63	30.61	2.87	0.633	1.074	24.30	41.60	9.36
		Rain-fed	32.53	30.27	32.40	3.49	0.373	0.282	22.60	43.20	10.78
