# Supplementary material for: QTL Mapping of Trichome Traits and Analysis of Candidate Genes in Leaves of Wheat (Triticum aestivum L.)
Source: Genes (Basel). 2023 Dec 27;15(1):42. doi: 10.3390/genes15010042 (PMC10815787; doi:10.3390/genes15010042)
Supplement: Supplementary file 1 [file genes-15-00042-s001.zip › Table 1.pdf]

Part	TD		TL	
	Irrigation	Rain-fed	Irrigation	Rain-fed
LT	57.076Aa	61.745Bb	37.967Aa	40.754**Aa
LC	60.813Aa	72.973**Aa	36.492Bb	36.843Bb
LB	46.218Bb	55.680**Bb	30.276Cc	32.634**Cc
E	35.785Bb	50.231**Bb	35.380Aa	37.264**Aa
M	62.535Aa	70.075**Aa	34.816Aab	36.717**Aab
NV	64.930Aa	72.653**Aa	34.540Ab	36.257**Ab
