# Supplementary material for: QTL Mapping of Trichome Traits and Analysis of Candidate Genes in Leaves of Wheat (Triticum aestivum L.)
Source: Genes (Basel). 2023 Dec 27;15(1):42. doi: 10.3390/genes15010042 (PMC10815787; doi:10.3390/genes15010042)
Supplement: Supplementary file 1 [file genes-15-00042-s001.zip › Table 2.pdf]

Table 2 Additive-effect QTLs for trichome density under two environments

QTL	Environment	Part	Positon (1)	Flanking marker	LOD[a]	PVE(100%)[b]	Add[c]
Qtd-2A-1	Rain-fed	LT-E	38	AX-110366793- AX-111505145	9.75	17.94	-10.09
		LT-NV			5.45	14.97	-9.63
Qtd-2A-2	Irrigation	LT-E	29	Xgwm275- Xwmc51	7.58	15.66	9.36
Qtd-2A-3	Irrigation	LC-E	34		8.84	14.929	-11.36
		LC-M			15.65	21.83	-16.71
		LC-NV			22.32	27.993	-17.94
Qtd-2A-4	Irrigation	LC-E	101	AX-111172526- AX-111745616	4.84	7.689	8.15
Qtd-2A-5	Irrigation	LC-M	20	Xcwm138.2- AX-110686688	5.64	6.92	9.52
Qtd-2A-6	Irrigation	LC-M	115	AX-109532624- AX-111046111	3.85	4.43	7.54
Qtd-2A-7	Irrigation	LC-NV	28	Xgwm425- Xpsp3088	8.79	9.923	10.68
Qtd-3A-1	Rain-fed	LT-M	96	AX-95653062- AX-95235020	4.69	12.37	-8.94
		LB-E	99		2.98	9.06	-5.05
		LB-M			3.30	9.24	-6.43
	Irrigation	LT-NV	96		5.10	11.93	-8.25
Qtd-3A-2	Rain-fed	LT-NV	91	AX-95207768- AX-95679334	6.18	16.67	-10.22
	Irrigation	LT-E	92		2.90	5.34	-5.52
		LC-NV			5.12	4.69	-7.40
Qtd-3A-3	Rain-fed	LC-E	90	AX-95232910- AX-95207768	4.31	9.20	-6.55
		LB-NV			4.427	10.12	-8.19
	Irrigation	LB-M	89		4.73	11.17	-8.16
Qtd-3A-4	Rain-fed	LC-M	100	AX-95235020- AX-95658735	4.09	10.15	-6.67
		LC-NV			3.76	10.39	-7.78
	Irrigation	LT-M			4.97	10.37	-8.26
Qtd-3A-5	Irrigation	LB-E	83	AX-110617585- AX-95072294	4.92	11.68	-8.07
Qtd-5A-1	Irrigation	LT-M	0	AX-109541070- AX-95630232	3.65	7.29	-6.95
Qtd-5A-2	Irrigation	LT-NV	20	AX-95659236- AX-109921026	3.07	6.89	-6.37
Qtd-6A-1	Rain-fed	LB-M	63	AX-110543147- Xpsp3071	2.57	7.34	5.90
Qtd-6A-2	Irrigation	LC-E	33	Xcwm162- AX-111619297	5.39	8.98	-9.28
Qtd-2B-1	Rain-fed	LC-NV	3	Xcwm529- AX-94481482	4.09	11.28	8.37
Qtd-2B-2	Irrigation	LT-M	2	AX-110555744- Xcwm529	2.66	5.86	6.39
Qtd-3B	Rain-fed	LT-E	47	AX-108735878- Xwmc231	4.93	9.56	-7.359
Qtd-5B-1	Rain-fed	LB-NV	99	Xgwm408- Xgwm604	2.577	5.97	6.27
Qtd-5B-2	Irrigation	LC-NV	133	AX-110945396- AX-109581522	2.65	2.41	5.30
Qtd-5B-3	Irrigation	LB-NV	249	AX-109455033- AX-108853192	2.76	8.60	7.08
Qtd-6B	Rain-fed	LT-E	75	EST138.1- Xwmc341	3.18	5.19	6.559
Qtd-7B	Rain-fed	LT-E	66	Xwmc269.1- Xgwm297	4.63	7.92	6.70
Qtd-1D	Irrigation	LC-NV	86	Xgwm337- AX-110521547	3.19	3.223	-6.08
Qtd-2D-1	Rain-fed	LC-E	95	AX-109879970- AX-111066402	6.93	15.24	-8.42
Qtd-2D-2	Rain-fed	LB-NV	89	AX-110744761- Xwmc453.1	4.47	10.64	-8.45
Qtd-2D-3	Irrigation	LB-M	116	AX-94735076- AX-111914966	3.53	8.13	-6.94
Qtd-3D	Rain-fed	LC-M	216	AX-109367171- AX-111568509	3.09	7.55	5.76
		LC-NV			3.51	9.60	7.49
Qtd-4D-1	Irrigation	LC-E	5	AX-111475478- AX-89654830	4.94	7.86	-8.25
		LC-M	7		5.77	6.913	-9.39
		LC-NV	8		6.67	6.42	-8.58
Qtd-4D-2	Irrigation	LB-E	2	AX-95659047- AX-109516054	4.27	9.99	-7.47
Qtd-6D	Rain-fed	LT-E	64	AX-108849732- AX-109779203	2.60	4.07	4.80
Qtd-7D-1	Rain-fed	LT-M	108	AX-111529990- AX-95631292	2.57	6.24	6.35
Qtd-7D-2	Rain-fed	LC-M	101	AX-95014724- AX-109507404	5.31	13.49	7.77

Note: (1) Position (cM) represents the distance to the first marker in the linkage group; (2) h^2(%) indicates the phenotypic variance explained by additive QTL; (3) A represent the additive effect. Positive value indicates the Hanxuan 10 allele having positive effect on the trait, and negative value represents Lumai 14 allele having positive effect .
