# Supplementary material for: QTL Mapping of Trichome Traits and Analysis of Candidate Genes in Leaves of Wheat (Triticum aestivum L.)
Source: Genes (Basel). 2023 Dec 27;15(1):42. doi: 10.3390/genes15010042 (PMC10815787; doi:10.3390/genes15010042)
Supplement: Supplementary file 1 [file genes-15-00042-s001.zip › Table 3.pdf]

Table 3 Additive effect QTLs for trichome length under two environments

QTL	Environment	Part	Position(1)	Flanking marker	LOD[a]	PVE(100%)[b]	Add[c]
Qtl-1A	Rain-fed	LC-E	73	Xcwm517- Xcwm516	2.50	6.03	-1.07
Qtl-2A-1	Rain-fed	LT-E	45	AX-94738148- Xgwm328	4.48	12.54	1.588
		LT-M	46		4.66	12.23	1.29
Qtl-2A-2	Rain-fed	LT-NV	37	AX-95684521- AX-111808444	3.18	8.53	1.05
Qtl-2A-3	Irrigation	LT-E	43	AX-94664242- AX-94738148	7.53	18.46	2.83
		LT-M	44		3.56	9.53	1.65
Qtl-2A-4	Irrigation	LT-NV	31	Xgwm249- Xwmc63	2.78	7.21	1.29
Qtl-2A-5	Irrigation	LB-E	95	AX-110402534- AX-94450655	2.57	6.66	1.04
Qtl-4A	Rain-fed	LC-M	53	AX-95145339- Xgwm610	3.68	8.09	1.00
Qtl-1B-1	Rain-fed	LT-E	118	Xwmc44- AX-108745931	2.95	8.16	-1.28
Qtl-1B-2	Rain-fed	LC-NV	42	AX-94658630- AX-94935185	2.54	9.18	-0.89
Qtl-2B-1	Rain-fed	LC-E	54	AX-108725943- AX-94940181	2.90	6.51	1.12
Qtl-2B-2	Rain-fed	LC-M	58	AX-108744217- AX-108728331	2.57	5.52	0.84
Qtl-3B	Irrigation	LT-M	34	AX-108749246- AX-111641535	2.91	7.23	1.44
Qtl-6B	Irrigation	LT-E	128	AX-111565328- AX-110521824	3.13	6.63	1.70
Qtl-7B-1	Rain-fed	LC-E	74	Xwmc269.1- Xgwm297	4.02	10.37	-1.39
Qtl-7B-2	Irrigation	LB-E	165	Xcwm466- AX-111065705	4.13	11.30	1.35
Qtl-7B-3	Irrigation	LB-NV	145	AX-108748008- AX-108801907	3.50	11.24	1.03
Qtl-2D	Irrigation	LC-M	90	Xwmc453.1- AX-109261081	3.39	10.79	-1.48
Qtl-3D	Irrigation	LB-M	161	AX-111086016- AX-111082209	3.96	8.49	1.08
Qtl-6D	Irrigation	LB-M	0	AX-111540806- AX-109555484	3.46	7.24	1.01
Qtl-7D-1	Rain-fed	LT-M	96	AX-95119219- Xgwm44	6.09	16.53	1.51
		LB-M			3.09	9.48	1.20
	Irrigation	LT-M	96		4.27	10.94	1.78
		LC-NV	95		5.66	16.01	1.83
Qtl-7D-2	Rain-fed	LT-NV	108	AX-111529990- AX-95631292	3.91	10.18	1.15
Qtl-7D-3	Rain-fed	LC-M	98	AX-109879968- AX-95014724	7.48	17.43	1.48
	Irrigation	LT-NV	100		6.43	17.70	2.05
		LB-NV	98		4.53	14.77	1.19
Qtl-7D-4	Rain-fed	LC-NV	94	AX-94850949- AX-95119219	3.88	14.14	1.12
	Irrigation	LT-E	93		4.81	10.60	2.17
Qtl-7D-5	Rain-fed	LB-E	103	AX-95014724- AX-109507404	2.67	8.34	1.09
	Irrigation	LC-E	101		2.99	9.13	1.60
		LB-M	103		4.94	10.65	1.21

Note: (1) Position (cM) represents the distance to the first marker in the linkage group; (2) h^2(%) indicates the phenotypic variance explained by additive QTL; (3) A represent the additive effect. Positive value indicates the Hanxuan 10 allele having positive effect on the trait, and negative value represents Lumai 14 allele having positive effect
