# Supplementary figures and images for: QTL Mapping of Trichome Traits and Analysis of Candidate Genes in Leaves of Wheat (Triticum aestivum L.)
Source: Genes (Basel). 2023 Dec 27;15(1):42. doi: 10.3390/genes15010042 (PMC10815787; doi:10.3390/genes15010042)

**a**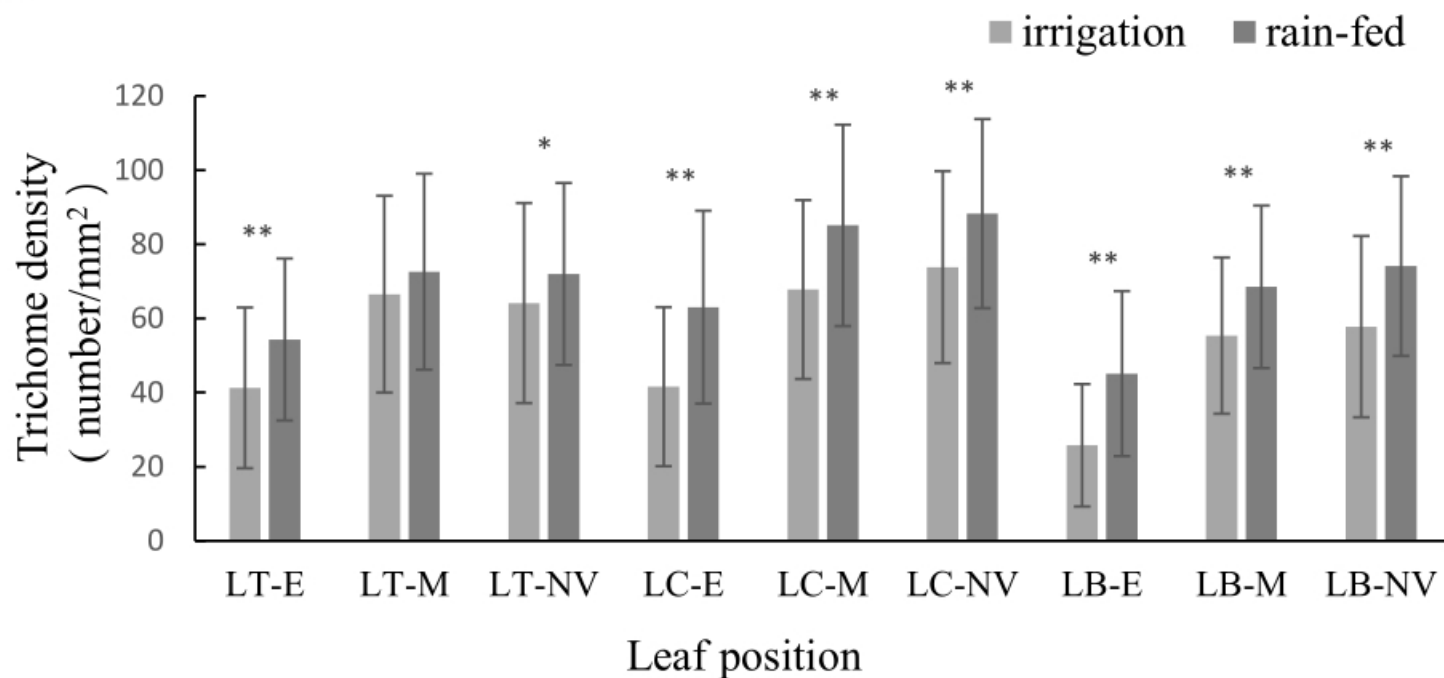**b**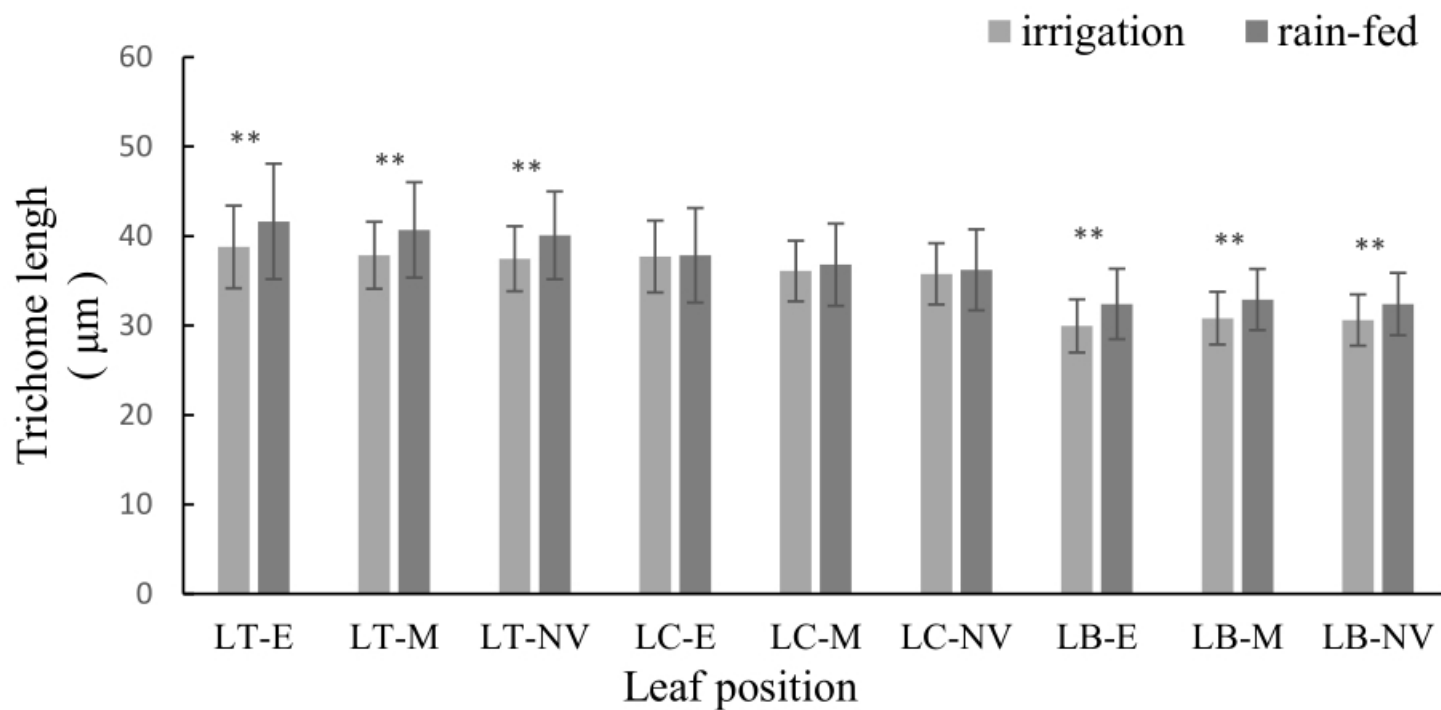

Supplement: Supplementary file 1 [file genes-15-00042-s001.zip › Figure 1.pdf]

**a**

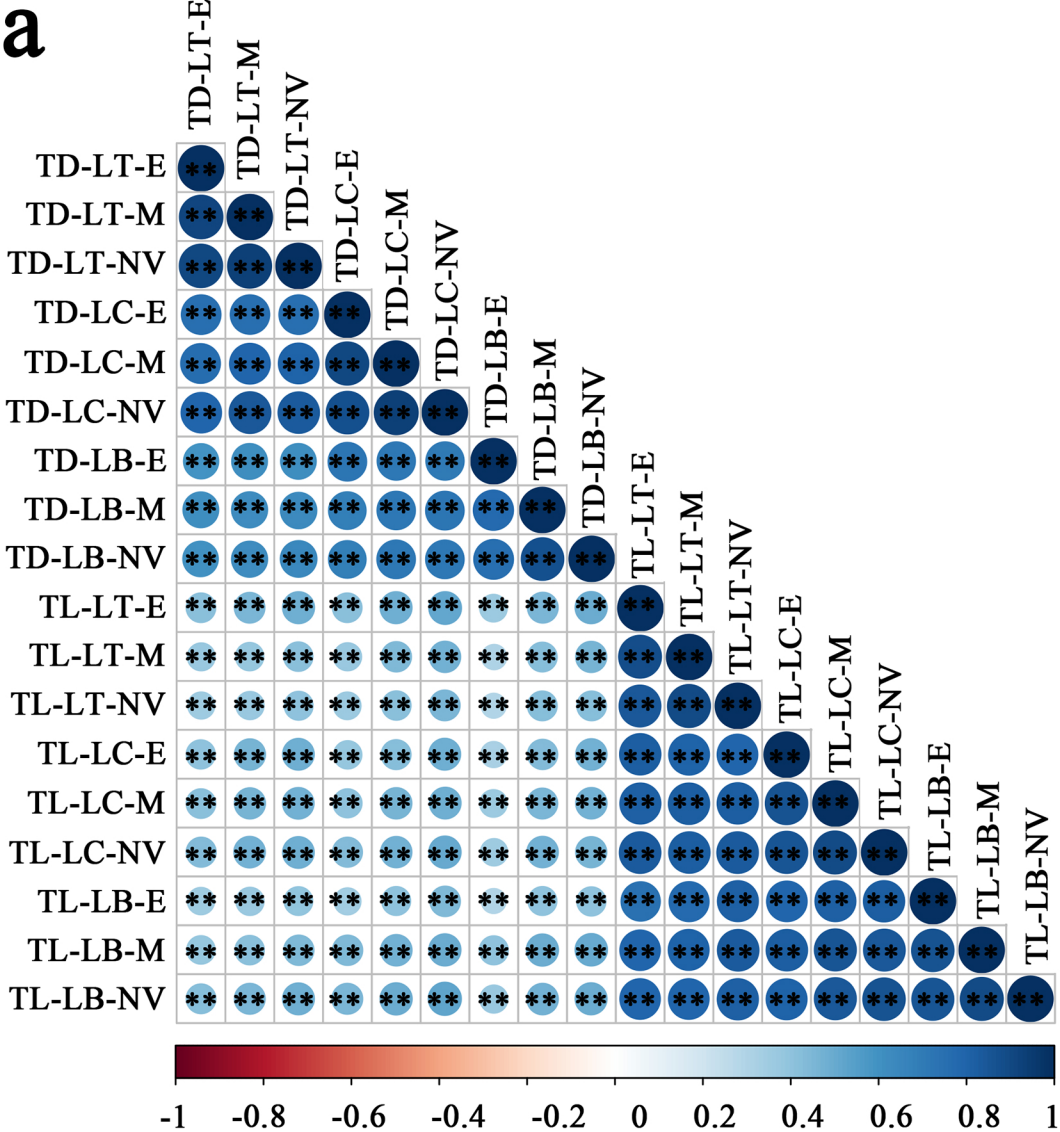**b**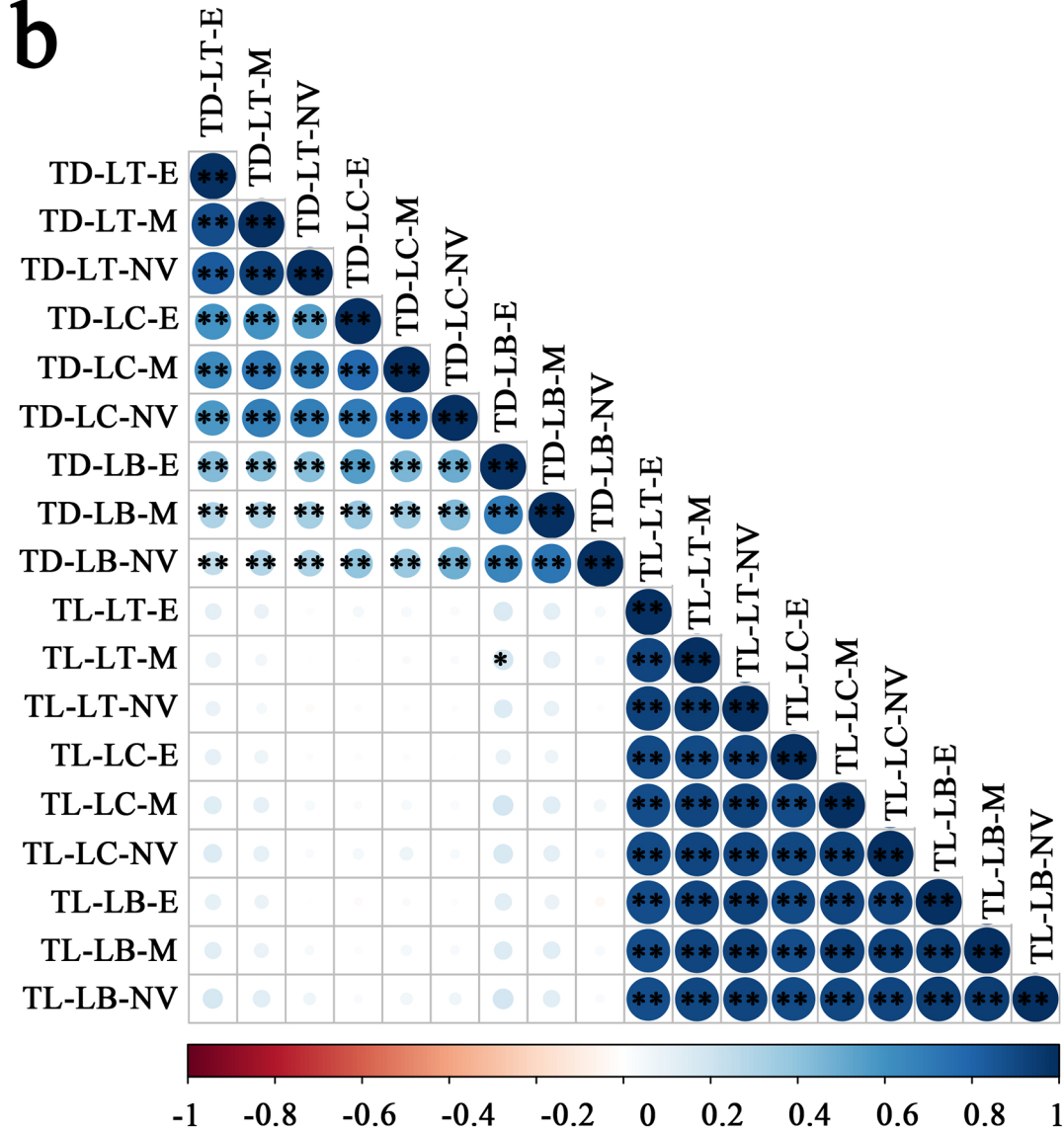

Supplement: Supplementary file 1 [file genes-15-00042-s001.zip › Figure 2.pdf]

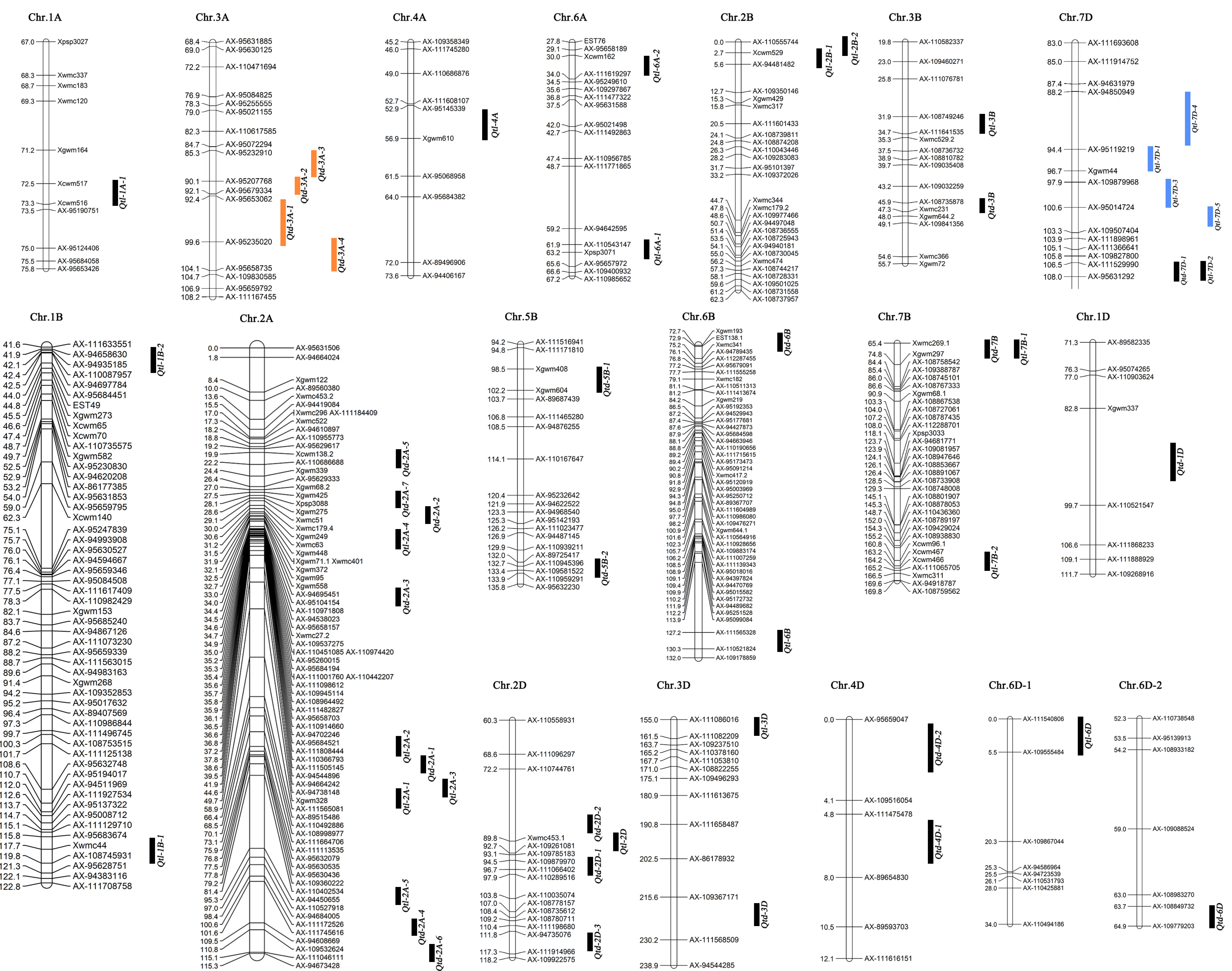

Supplement: Supplementary file 1 [file genes-15-00042-s001.zip › Figure 3.pdf]

(a)

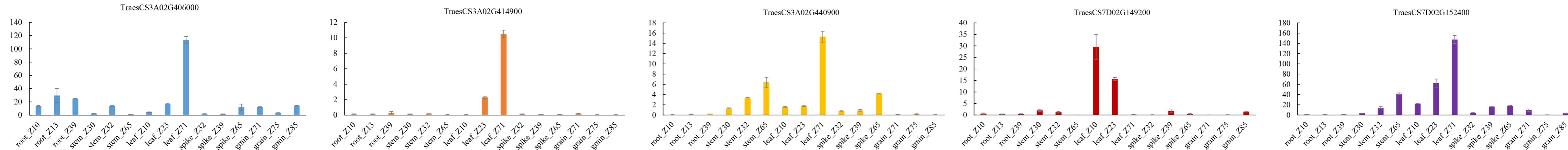

(b)

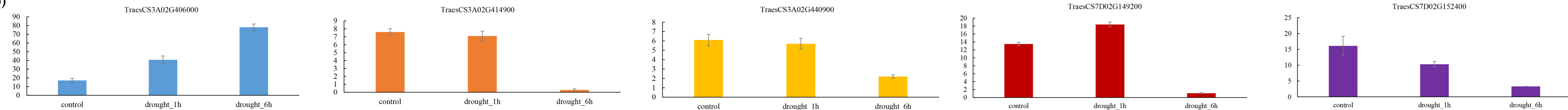

Supplement: Supplementary file 1 [file genes-15-00042-s001.zip › Figure 4.pdf]

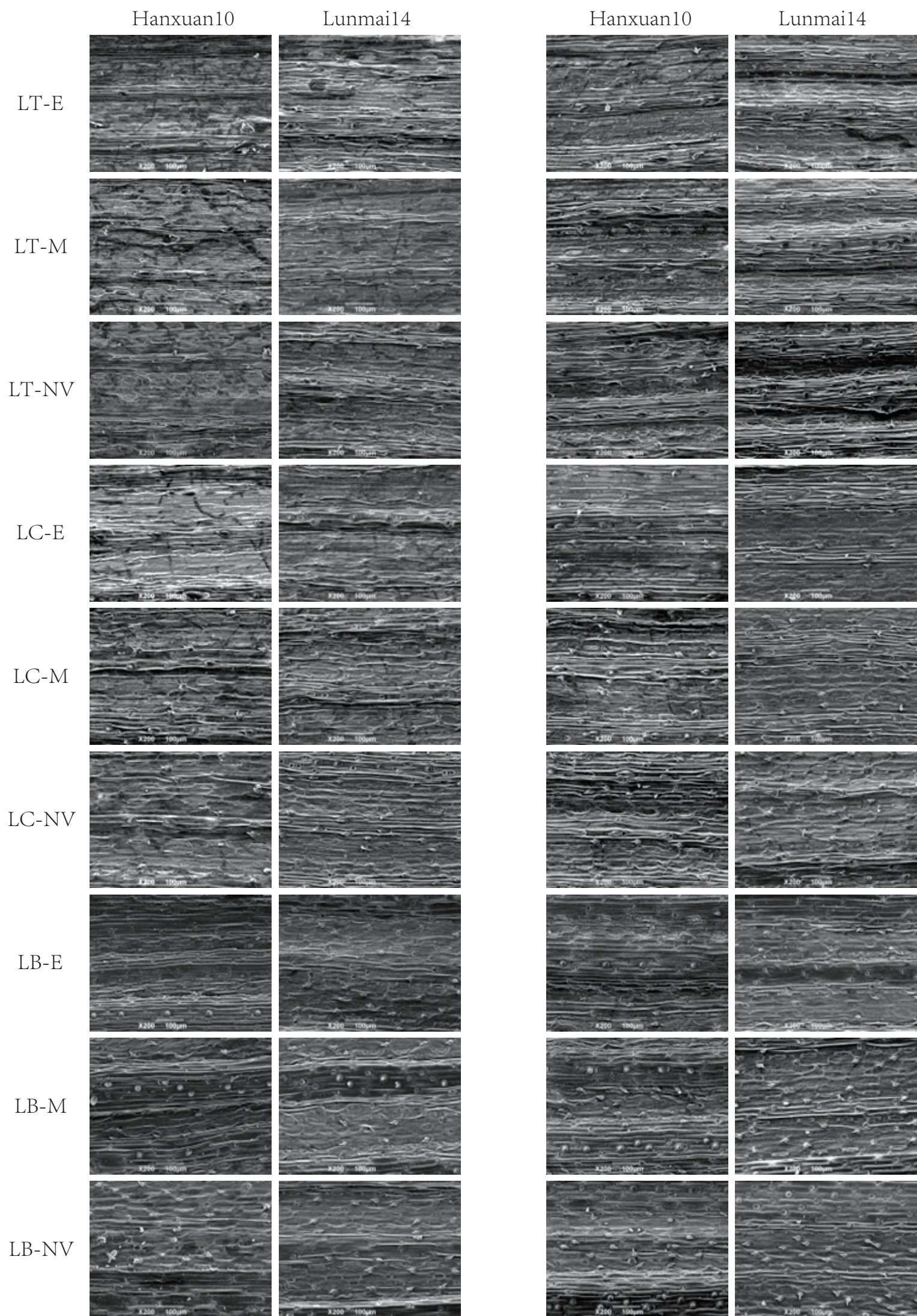

WW

DS

Supplement: Supplementary file 1 [file genes-15-00042-s001.zip › Supplementary Figure 1.pdf]
